# The Role of Microbiology at the 1-Month Surveillance Bronchoalveolar Lavage in the Identification of Complications in the First Year After Lung-Transplantation—A Retrospective Single-Center Experience

**DOI:** 10.3390/life15091462

**Published:** 2025-09-17

**Authors:** Rocco Francesco Rinaldo, Antonio Curtoni, Mattia Verardo, Silvia Zaffina, Nour Shbaklo, Francesca Sidoti, Francesco Giuseppe De Rosa, Silvia Corcione, Massimo Boffini, Matteo Marro, Cristina Costa, Paolo Solidoro

**Affiliations:** 1 Division of Respiratory Medicine, Cardiovascular and Thoracic Department, AOU Città Della Salute e Della Scienza di Torino, 10126 Torino, Italy; roccofrancesco.rinaldo@unito.it (R.F.R.); mattia.verardo@gmail.com (M.V.); paolo.solidoro@unito.it (P.S.); 2 Medical Sciences Department, University of Turin, 10126 Torino, Italy; silvia.zaffina@unito.it; 3 Division of Virology, Department of Laboratory Medicine, AOU Città Della Salute e Della Scienza di Torino, 10126 Torino, Italy; antonio.curtoni@unito.it (A.C.); francesca.sidoti@unito.it (F.S.); cristina.costa@unito.it (C.C.); 4 Department of Public Health and Paediatrics, University of Turin, 10126 Turin, Italy; 5 Unit of Infectious Diseases, Department of Medical Sciences, University of Turin, 10149 Torino, Italy; francescogiuseppe.derosa@unito.it (F.G.D.R.); silvia.corcione@unito.it (S.C.); 6 Cardiac Surgery Division, Cardiovascular and Thoracic Department, AOU Città Della Salute e Della Scienza di Torino, 10126 Torino, Italy; massimo.boffini@unito.it (M.B.); matteo.marro1@gmail.com (M.M.); 7 Surgical Sciences Department, University of Turin, 10126 Torino, Italy

**Keywords:** bronchoalveolar lavage, lung-transplantation, microbiology, surveillance, COPD

## Abstract

Acute rejection and infections are the most frequent complications in the first year after lung transplantation, often representing relevant causes of death. There is still no consensus on the ideal strategy for preventing these events, with a still open debate on active bronchoscopic surveillance protocols vs. clinically mandated ones. The aim of our single-center exploratory study was to evaluate retrospectively the role of microbiology at bronchoalveolar lavage (BAL) at the first month from transplantation in asymptomatic patients in relation to the development of complications up to 12 months from surgery. We collected data from 28 patients who underwent surveillance bronchoscopies according to our center protocol (transbronchial biopsies and BAL at months 1, 4, 8, 12, 18, and 24 post-transplantation) who had a 12-month follow-up. The inclusion criterion was the absence of infiltrates at 1-month post-transplantation chest CT. We excluded patients transplanted due to suppurative diseases of the lung to minimize the pre-transplantation risk factors for infection. We also assessed differences in complications according to the underlying disease. We enrolled 15 patients with interstitial lung diseases (ILDs) and 13 with chronic obstructive pulmonary disease (COPD). Of the 28 patients, 11 had a positive BAL for bacteria. Patients with a positive BAL developed a higher number of pulmonary infectious complications (odds ratio of 18.33, *p*-value = 0.013 at regression model), with a near significance for moderate–severe pulmonary infections (odds ratio 4.8, *p*-value = 0.061). We did not find a significant correlation with rejection, cytomegalovirus reactivation, or pseudomembranes. We did not find differences in the rates of complications when grouping subjects according to pre-transplantation disease. Our results suggest a possible role for BAL positivity for bacteria in asymptomatic patients at surveillance bronchoscopy in predicting the development of future infections, warranting a tailored follow-up of patients that considers this data. Larger, multicentric studies are needed to explore and confirm the utility of our findings.

## 1. Introduction

Lung transplantation has become an internationally acknowledged therapeutic approach for the management of end-stage pulmonary diseases, offering an expected improvement in the survival and quality of life of the recipients [1]. Idiopathic and non-idiopathic interstitial diseases (ILDs), chronic obstructive pulmonary disease (COPD), and cystic fibrosis (CF) represent the most frequent indications for lung transplantation [2].

The maintenance of graft function and the recipient’s overall well-being require vigilant surveillance and a delicate equilibrium between the benefits and potential harms of prolonged immunosuppressive therapy [3]. The most frequent causes of complications leading to death in the first year after lung transplantation are non-cytomegalovirus (CMV) infections and graft failure [4,5], with infections being the most likely cause of new infiltrates after 30 days from transplantation [6].

Moreover, infections can play a role in contributing to graft failure itself [7]. To prevent the occurrence of such complications after lung transplantation, a keen clinical and functional (through pulmonary function tests) monitoring is fundamental, as well as through invasive methods such as surveillance bronchoscopy, bronchoalveolar lavage (BAL), and transbronchial biopsy (TBB) of the lung allografts. Mixed opinions can be found in the literature regarding the role of pre-scheduled active surveillance bronchoscopy compared to a clinically mandated one, with no definitive guidelines [8,9]. An acknowledged benefit of active surveillance is the possibility of identifying asymptomatic infections or early-stage phases of rejection [10,11]. Nevertheless, some pre-transplantation risk factors for infections have been identified: conditions such as CF and suppurative diseases of the lung, diabetes mellitus, obesity or malnutrition, viral hepatitis, HIV infection, latent tuberculosis infection (LTBI), colonization with multidrug-resistant (MDR) bacteria, chronic kidney disease, and the requirement for mechanical ventilation are all recognized as features that can contribute to the development of bacterial infections after transplantation [12].

The primary objective of our study was to evaluate whether, in a cohort of lung transplantation recipients without major risk factors (i.e., excluding those with suppurative lung diseases) and with normal chest computed tomography (CT) findings at one month post-transplantation, microbiological results for bacteria from bronchoalveolar lavage (BAL), obtained through an active bronchoscopic surveillance strategy, could help identify patients at higher risk of developing complications within the first year after transplantation. BAL positivity was also studied in relation to the severity of pulmonary infectious complications. Moreover, we wished to assess, as an exploratory outcome, whether differences could be found in this sense according to the pathology that led to transplantation.

## 2. Materials and Methods

### 2.1. Study Design

The data analyzed in this study were derived from clinical, microbiological, functional, and radiological assessments of patients who underwent lung transplantation at the Lung Transplantation Center at the City of Health and Science of Turin, Molinette Hospital, from April 2022 to April 2024, for patients with a follow-up of at least 12 months. Exclusion criteria were a pre-transplantation diagnosis of suppurative disease of the lung, including CF. Patients presenting with radiological infiltrates on the 1-month post-transplantation surveillance CT scan were excluded [13].

### 2.2. Data Collection

Clinical data were collected from the dedicated software database (ITR02, Regional Transplantation Information System of Piedmont and Aosta Valley, Oracle Fusion Middleware). Collected data included the primary diagnosis, biometric and microbiological data, pathological reports, functional and radiological findings, and clinical records from the time of transplantation eligibility evaluation through the entire post-transplantation follow-up period. Data was collected on transplantation date (T0) and at 1–12 months post-transplantation (T1–12).

BAL samples were collected during routine surveillance bronchoscopies in accordance with the transplantation center’s protocol and analyzed at the microbiology laboratory of the center. Flexible video–bronchoscopes were used, and three 50 mL aliquots of saline solution were instilled and aspirated from subsegmental bronchi in target areas (middle lobe or lingula) [10].

### 2.3. Complications

BAL samples with >10,000 copies of CMV DNA and serum samples with >100,000 copies of CMV DNA were considered clinically significant. Lung function was evaluated between time intervals and classified as improved or worsened if FEV1 changed by more than 10% from the best FEV1.

Acute rejection was classified according to the histological criteria established by the International Society for Heart and Lung Transplantation [14]. We also collected data on the detection of pseudomembranes at direct visualization during the bronchoscopies [8]. All BAL samples underwent quantitative PCR for microbial DNA/RNA detection, culture testing, and microscopy for mycobacteria. A threshold of 25.000 CFU was seen as microbiologically significant.

Pulmonary infectious complications within the first year were stratified by severity [15]:

- Mild: Low-to-moderate microbial load on BAL with no or initial radiological alterations and absence of symptoms.
- Moderate: Medium-to-high microbial load with initial symptoms and radiological findings, but without respiratory failure.
- Severe: Microbial isolation associated with respiratory failure and radiological abnormalities.

### 2.4. Surveillance Protocol

The transplantation center’s surveillance protocol for early detection of rejection and infectious complications includes scheduled transbronchial biopsies and BAL at months 1, 4, 8, 12, 18, and 24 post-transplantation [16]. Each BAL is tested for CMV and EBV DNA, galactomannan and beta-D-glucan antigens, and both general and mycobacterium-specific cultures. Each procedure was performed by the same team of trained bronchoscopists. Routine follow-up visits include pulmonary function tests (PFTs) and blood tests, including immunosuppressant levels, serum CMV and EBV DNA, galactomannan antigen, and beta-D-glucan.

#### 2.4.1. Antimicrobial Prophylaxis and Immunosuppressive Protocols

Post-transplantation antimicrobial prophylaxis was carried out with Vancomycin guided by blood levels and Ceftazidime 1 gr tid, both for 10–15 days, with regard to antibiotic therapy in the ICU, and then with trimethoprim-sulfamethoxazole, antiviral (acyclovir and valganciclovir), and topical antifungal (oral suspension) in the long term; in addition, CMV immunoglobulin therapy was administered according to body weight.

The initial immunosuppression protocol at our facility involved intravenous corticosteroid therapy (methylprednisolone) followed by oral therapy (prednisone), combined with a calcineurin inhibitor (e.g., cyclosporine or tacrolimus) and an antimetabolite (e.g., mycophenolate). As an alternative to mycophenolate, after 6 months, an m-tor inhibitor (e.g., everolimus) was considered in cases of functional decline or documented rejection on follow-up transbronchial biopsies. The dosage of the various drugs has been adjusted based on periodic blood level testing. 

#### 2.4.2. Statistical Analysis

Categorical variables were described using frequencies and percentages. Continuous variables were reported as mean and standard deviation or median and interquartile range (IQR) according to the distribution. Differences in categorical variables were analyzed using Chi-square or Fisher’s exact tests. For continuous variables, depending on data distribution, Student’s *t*-test or Mann–Whitney U test was applied. Statistical analyses were performed using Jamovi software 2.6.44. Binomial regression was used to test if the BAL positivity significantly predicted complications. A *p*-value of <0.05 was considered statistically significant.

## 3. Results

During the study period, we evaluated 37 patients who underwent lung transplantation at our center: 2 (5%) were excluded due to early complications and death, 5 (13%) were excluded due to a pre-transplantation diagnosis of suppurative disease of the lung, and 2 (5%) were excluded due to the presence of lung infiltrates at the chest CT prior to the 1-month bronchoscopy. None of the patients enrolled had signs of previous colonization on the respiratory tract samples obtained in the pre-transplantation workup.

Thus, we collected data from 28 patients, distributed as 11 (39%) females and 17 (61%) males. In total, 13 (46%) had a pre-transplantation diagnosis of COPD/emphysema, and 15 (54%) had ILDs. The median age at transplantation was 62 years (IQR 59–64). Mortality at 12 months was reported in 1 patient (3.5% of the population), which was due to sepsis of an abdominal origin.

Complications recorded were acute rejection in 21 patients (75%) within the first 12 months, anastomotic pseudomembranes in 20 (71%), and CMV DNA reactivation in 8 (28%) patients, Table 1.

Pulmonary infectious complications were mild in 2 (7%) patients, moderate in 12 (42%), and severe in 2 (7%), Table 2. Extrapulmonary infectious complications were not seen in 24 (85%) patients; however, urinary tract infections were seen in 2 (7%), peritonitis in 1 (3%), and sepsis of unknown origin in 1 (3%).

Four patients underwent a single lung transplantation; all of them resulted in negative BAL for bacteria in the first month. Two developed a moderate pulmonary infectious complication during the follow-up. At 1-month post-transplantation, 17 patients had negative BAL results in standard culture tests, while 11 patients were positive for bacterial agents in significant CFU numbers, despite a lack of clinical, radiological, or laboratory signs of pneumonia.

In the BAL-positive group, 2 donors were reported for infections (one metycillin-sensitive *S. Aureus*–MSSA–endocarditis and one bronchial aspirate *E. bugandensis*), while 2 donors in the BAL-negative group were reported: one for bronchoaspirate positivity for MSSA and *E. cloacae* and the second for MSSA on bronchoaspirate. This data was an indication for modifying post-transplantation anti-microbial prophylaxis accordingly. None of the 2 BAL-negative patients developed an infectious complication in the follow-up, while both BAL-positive patients did (one mild and one severe).

Among the 11 BAL-positive patients, 4 developed ventilator-associated pneumonia (VAP) during the ICU stay (one *A. baumannii* isolate, the other three were treated empirically with broad-spectrum therapy) and were treated accordingly, prior to the first month of BAL. The four patients all developed an infectious complication in the follow-up (1 mild, 2 moderate, 1 severe). Among the 17 BAL-negative patients, 2 developed infective events in the first month: 1 VAP due to MRSA and 1 *K. pneumoniae* sepsis. They both developed infectious pulmonary complications in the follow-up.

We did not find differences in BAL-positive and -negative patients in the number of days in ICU (5 (3–9) vs. 4 (3–7); *p*-value = 0.487) and hospital stay (34 (29–56) vs. 42 (33–73); *p*-value = 0.517), respectively.

Patients with BAL positivity at 1-month post-transplantation developed significantly more pulmonary infections within 12 months (*p* = 0.004), Table 1. Considering the significant result, a regression model was run, showing 38.5% variability of pulmonary infectious complications and an odds ratio (OR) of 18.33 (CI 95% 1.87–180; *p*-value = 0.013).

A significant association between the severity levels (none, mild, moderate, and severe) and BAL results was reported, *p* = 0.009, Table 2. Complications were also reclassified into none to mild and moderate to severe; the univariate analysis yielded a nearly significant result, with a *p*-value = 0.053. However, the binary regression model was not significant, showing a 17% variability of pulmonary infectious complications, with OR 4.8 (CI 95% 0.93–25.6; *p* = 0.061).

A marginal association was also noted between BAL positivity and the presence of pseudomembranes (*p* = 0.066), Table 1. No significant associations were found between BAL positivity and rejection, extrapulmonary infections, or CMV reactivation. The only patient who died because of septic shock caused by an abdominal infection had a negative BAL at the first surveillance bronchoscopy.

Surveillance of symptomatic BAL findings included the following: *S.aureus* in 3 (27%) and *P. aeruginosa* in 3 patients (27%), *Enterobacter* spp. in 1 patient (9%), and *K. pneumoniae* in 2 (18%) and *S. maltophilia* in 2 patients (18%). Severe infectious complications included the following: 3 (10%) cases of sepsis (unspecified pathogen, *K. pneumoniae*, *E. coli*), 3 (10%) cases of pneumonia (*P. aeruginosa*, *S. maltophilia*, unspecified), and 1 (3%) urinary tract infection with *K. pneumoniae*. Table 3 summarizes the isolates, including antibiogram and whether a treatment was carried out.

We also evaluated, in the whole sample, whether the presence of endobronchial anastomotic pseudo membranes, identified during surveillance bronchoscopies in the post-transplantation period, affected PFTs at time points T1, T4, T8, and T12 when available, with no significant differences detected, Table 4. We also did not find any difference regarding the detection of pseudomembranes and frequency of rejection or a higher incidence of moderate to severe pulmonary infections.

Comparing COPD and ILDs populations, the incidence of infectious pulmonary complications was not statistically significantly different between the groups (Table 5). No statistical significance was found regarding differences in the incidence of rejection or need for high-dose steroid therapy. No differences emerged between the two comparison groups in terms of the frequency of pseudomembrane detection by bronchoscopy as well.

No statistically significant differences were found between the COPD and ILDs populations in terms of post-lung transplantation hospitalization days (Table 6).

Regarding CMV reactivations in transplanted subjects, no statistically significant differences were found between patients with COPD and those with ILDs. CMV reactivation (>10,000 copies in BAL at T4–T12) showed no significant difference between COPD and ILD patients (Table 7).

## 4. Discussion

The main results from this exploratory, hypothesis-generating study on the role of microbiology in surveilling BAL at the first month post-lung transplantation in patients with no signs of pulmonary infiltrates and no pre-transplantation risk factors for infections are as follows: (1) there is a significant correlation between the positivity of the 1-month surveillance BAL for bacteria and the development of pulmonary infectious complications in the first year from lung transplantation; (2) there is a borderline association between the positivity of the 1-month surveillance BAL and the development of moderate–severe pulmonary infectious complications; and (3) there is no difference in terms of complications in the first year between patients who underwent lung transplantation due to COPD or ILDs.

The first month post-transplantation is characterized by the combination of immunosuppression (and/or defects in immune function) and factors such as surgical complications (including technical issues, type of transplantation), donor-derived infections, preexisting recipient infections, aspiration, and hospital environmental exposure-related infections [17,18]. This overall immunological state might explain our findings, as we obtained positive cultures for bacteria in patients with no radiological signs compatible with pneumonia, even after 30 days post-surgery. In our study, patients presenting a BAL positivity for bacteria at the first surveillance bronchoscopy showed a significantly higher rate of infectious complications up to 12 months from transplantation (with a trend for moderate–severe pulmonary infections). This is in line with the literature, which suggests, as underlying causes of the latter transplantation infections, the activation of latent, relapsed, residual, opportunistic infections; exposure to community-related pathogens; and the immunological role of graft rejection and the immunosuppressive treatment levels required to treat/prevent it [17,19].

The rate of infectious complications we report is higher than that reported in the literature. Gagliotti et al. reported a share of 47% infectious complications in lung transplantation recipients in the first 6 months after transplantation [20]. However, our sample is composed of a selected population, from which we excluded subjects who died before completing the 12-month follow-up or presenting signs of early complications.

While the role of some asymptomatic viral infections is more clearly described in the literature, with CMV, for example, often requiring preemptive therapy [21], less is known about asymptomatic bacterial positivity on lower airway specimens [22,23]. In our sample, we identified mainly Gram-negative pathogens in the first month of BAL (63%), while Staphylococcus aureus accounted for the remaining Gram-positive isolates (37%). This is in line with the current literature, with most of the isolates including Pseudomonas aeruginosa and Klebsiella pneumoniae and a reduced rate of Gram-positives, particularly methicillin-resistant Staphylococcus aureus [17,18,24]. These data also confirm the high incidence and relevance of such pathogens, which have the potential to quickly escalate to fatal infections in solid organ transplantation recipients [20], beyond being a potential threat to other patients, even in asymptomatic patients.

An indirect indication of the importance of early-phase post-transplantation and of surveillance bronchoscopy comes from the study of Takizawa et al., who found that the information obtained in the first two procedures (two and six weeks from transplantation) impacted clinical management, while the effect was diluted in the following procedures (up to 12 months) [25].

We did not find any difference in terms of acute rejection according to the first month of BAL microbiology. Generally, infections are considered a potential risk factor for ACR, although among several others, such as subtherapeutic immunosuppressant drug levels, aspiration, and gastroesophageal reflux [26,27]. However, in our study, we assessed the role of BAL results in a cohort of patients with no radiological signs of lung infection, thus not overtly infected. In addition, a more solid role of infections is acknowledged in the development of long-term graft dysfunction, such as chronic lung allograft dysfunction (CLAD), rather than short-term dysfunction. [19,28].

Our study confirms that there is no difference in post-transplantation outcomes between the two underlying conditions we examined (COPD and ILDs), especially in the short to medium term [29,30]. Particularly, some recent registry data on lung transplantation patients between 2016 and 2020 showed similar rates of post-transplantation complications in a population of patients affected by COPD and IPF, respectively, 51% and 47.5% [31,32]. In both populations, rejection was the most reported complication (24% vs. 25%), followed by infections (13.4% vs. 11%). In addition, a direct comparison between the IPF and non-IPF populations did not reveal significant differences in the rate of complications in the post-transplantation period [32]. However, specifically for our study, the comparison between the COPD and ILD groups was limited by the small sample size, and the absence of observed differences may be reflective of the limited sample rather than equivalence between the groups. Of note, the literature shows how fibrotic patients globally have a shorter survival post-transplant and a possibly higher rate of chronic allograft dysfunction in the long term [29,33]. Nevertheless, Paglicci et al. reported a lower rate of bacterial infections in COPD patients in their single-center retrospective study [18].

Our exploratory results suggest a possible role for the first month BAL microbiology in tailoring the follow-up of lung transplantation patients, independently of the underlying cause of transplantation, even in the absence of a suppurative disease of the lung. Larger prospective studies are warranted to explore this area. Nevertheless, the debate on the utility of surveillance bronchoscopy is still open, with authors demonstrating a lack of true benefits. For example, Valentine et al. examined 240 TBB/BALs obtained either from clinically mandated (84 procedures) or surveillance (156 procedures, of which 54 were scheduled and 102 were from clinical indication) protocols in their single-center study. No advantage in terms of detection of acute rejection, infection, or bronchiolitis obliterans-free survival was identified. Complications in the true surveillance subgroup ranged from fever, infiltrates, and emesis to pneumothorax requiring hospitalization [34], confirming the generally acknowledged risk of active surveillance protocols. On the other hand, the possible adoption of a lower threshold for performing the procedures in patients managed with no bronchoscopic surveillance protocol was proposed to explain a similar number of procedures performed between the two strategies in different studies [35,36,37].

This study has several limitations that must be acknowledged. Firstly, its monocentric and retrospective nature limits the generalizability of our results. In addition, the small sample size might have limited our statistical power, with a risk of overestimation of effect sizes and increasing the likelihood of type I error. We tried to select our sample excluding patients with a recognized risk factor for post-transplantation infections, such as a diagnosis of suppurative disease of the lung; however, it must be noted that we had no access to the complete pre-transplantation microbiology data on the procured organs, which are reported only in case of significant isolates.

## 5. Conclusions

Post-lung-transplantation patient management is still a subject of uncertainty, with most of the related literature based on experts’ opinions and single-center retrospective studies, with different epidemiology and local experience-based protocols, among centers. Particularly, the role of active bronchoscopic surveillance vs. clinically mandated one is a matter of debate. The results of our exploratory study suggest a possible role for BAL positivity for bacteria in asymptomatic patients at surveillance bronchoscopy in predicting the development of future infections up to 12 months from transplantation, warranting a tailored follow-up of patients that considers this data. However, we did not find differences in this sense between patients who underwent transplantation due to COPD or ILDs, within the limits of a possibly underpowered sample. Larger, multicentric studies are needed to explore and confirm the utility of our findings.

## Figures and Tables

**Table 1:** Pulmonary infection complication and BAL.

Variable	Negative BAL n = 17 (60%)	Positive BAL n = 11 (39%)	Total n = 28 (100%)	*p*-Value
Pulmonary Infective complications	6 (35%)	10 (90%)	16 (57%)	0.004
Pseudomembrane	10 (58%)	10 (90%)	20 (71%)	0.066
Acute rejection	12 (70%)	5 (45%)	17 (60%)	0.183
CMV reactivation at T1	2 (11%)	0	2 (7%)	0.765
Extrapulmonary infections	15 (88%)	9 (81%)	24 (85%)	0.635

**Table 2:** Pulmonary complication severity on T1.

Complication Type	Negative BAL n = 17 (60%)	Positive BAL n = 11 (39%)	Total n = 28 (100%)	*p*-Value
None	11 (64%)	1 (9%)	12 (42%)	0.009
Mild	0	2 (18%)	2 (7%)	
Moderate	6 (35%)	6 (54%)	12 (42%)	
Severe	0	2 (18%)	2 (7%)	
Associated:				
None to mild	11 (64%)	3 (27%)	14 (50%)	0.053
Moderate to severe	6 (35%)	8 (72%)	14 (50%)	

**Table 3:** Microbiological findings in BAL samples.

Patient	Isolate	Sensitive	Resistant	Treatment	Reason for Treatment	Pulmonary Infection Within 12 M
1	*E. cloacae*	aminoglycoside monobactam	beta-lactam carbapenem FQ	CAZ-AVI aztreonam	previous pneumonia	moderate
2	*P. aeruginosa*	beta-lactam	FQ	none	-	moderate
3	*P. aeruginosa*	muti-sensitive	none	none	-	moderate
4	*P. aeruginosa*	multi-sensitive	none	none	-	moderate
5	*S. maltophilia*	TMP/SMX	none	TMP/SMX	ID consultation	moderate
6	*S. maltophilia*	TMP/SMX	none	meropenem	ID consultation	none
7	*S. aureus*	methicillin	none	cefexime prulifloxacin	concomitant rejection and steroid bolus	mild
8	*K. pneumoniae* ESBL+	pip-tazo	FQ beta-lactam (except pip-tazo)	meropenem	High CFU	Severe
9	*K. pneumoniae* ESBL+	aminoglycoside carbapenems	FQ beta-lactam	none	-	severe
10	*S. aureus*	tetracycline linezolid	methicillin FQ	linezolid	concomitant rejection and steroid bolus	mild
11	*S. aureus*	tetracycline linezolid	methicillin FQ	linezolid	ID consultation	moderate

Legend: FQ: fluoroquinolone, CAZ-AVI: ceftazidime–avibactam, ID: infectious diseases, TMP/SMX: trimethoprim/sulfamethoxazole, pip-tazo: piperacillin-tazobactam, CFU: colony-forming uni.

**Table 4:** Pseudomembrane and functional trend of respiratory function.

Time	Worsening n = 9	Stable n = 26	Improved n = 19	*p*-Value
T4-T1	3 (33%)	6 (23%)	10 (52%)	0.402
T8-T4	1 (11%)	13 (50%)	4 (21%)	0.643
T12-T8	5 (55%)	7 (27%)	5 (26%)	0.287

**Table 5:** COPD vs. ILD.

Variable	COPD n = 13 (47%)	ILD n = 15 (53%)	Total n = 28 (100%)	*p*-Value
Acute rejection	10 (77%)	11 (73%)	21 (75%)	0.535
Steroids in bolus	5 (38%)	9 (60%)	14 (50%)	0.256
Pseudomembrane	9 (69%)	11 (73%)	20 (71%)	0.811
CMV quantiferon	5 (38%)	9 (60%)	14 (50%)	0.255
Pulmonary complication:				0.705
Mild	7 (53%)	7 (46%)	14 (50%)	
Moderate-severe	6 (46%)	8 (53%)	14 (50%)	

**Table 6:** Time for admission in COPD and ILD patients.

Variable	COPD	ILD	*p*-Value
Length of stay	39 ± 15	50 ± 28	0.333
ICU days	5 ± 3	11 ± 14	0.155
Age at transplantation	61 ± 5	61 ± 3	0.594

**Table 7:** CMV DNA frequency in BAL.

DNA in BAL	COPD n = 13 (47%)	IPF n = 15 (53%)	Total n = 28 (100%)	*p*-Value
T1				
>10 k	1 (8%)	1 (6%)	2 (7%)	0.916
T4				0.189
Negative	5 (38%)	7 (47%)	12 (42%)	
>10 k	5 (38%)	2 (13%)	7 (25%)	
>100 k	2 (15%)	6 (40%)	8 (28%)	
T8				0.073
Negative	6 (46%)	6 (40%)	12 (42%)	
>10 k	4 (30%)	1 (6%)	5 (18%)	
>100 k	1 (8%)	6 (40%)	7 (25%)	
T12				0.023
Negative	8 (61%)	7 (47%)	15 (53%)	
>10 k	2 (15%)	2 (13%)	4 (14%)	
>100 k	2 (15%)	2 (13%)	4 (14%)	

Legend: K = thousand.

## Data Availability

The original contributions presented in this study are included in the article. Further inquiries can be directed to the corresponding author(s).
